# Molecular neurodegeneration: basic biology and disease pathways

**DOI:** 10.1186/1750-1326-9-34

**Published:** 2014-09-23

**Authors:** Robert Vassar, Hui Zheng

**Affiliations:** Northwestern University, Feinberg School of Medicine, Chicago, IL USA; Baylor College of Medicine, Houston, Tx USA

## Abstract

The field of neurodegeneration research has been advancing rapidly over the past few years, and has provided intriguing new insights into the normal physiological functions and pathogenic roles of a wide range of molecules associated with several devastating neurodegenerative disorders, including Alzheimer’s disease, Parkinson’s disease, amyotrophic lateral sclerosis, frontotemporal dementia, Huntington’s disease, and Down syndrome. Recent developments have also facilitated initial efforts to translate preclinical discoveries toward novel therapeutic approaches and clinical trials in humans. These recent developments are reviewed in the current Review Series on "***Molecular Neurodegeneration****: Basic Biology and Disease Pathways*" in a number of state-of-the-art manuscripts that cover themes presented at the Third International Conference on Molecular Neurodegeneration: "*Basic biology and disease pathways*" held in Cannes, France, September, 2013.

## Text

**Figure 1:**
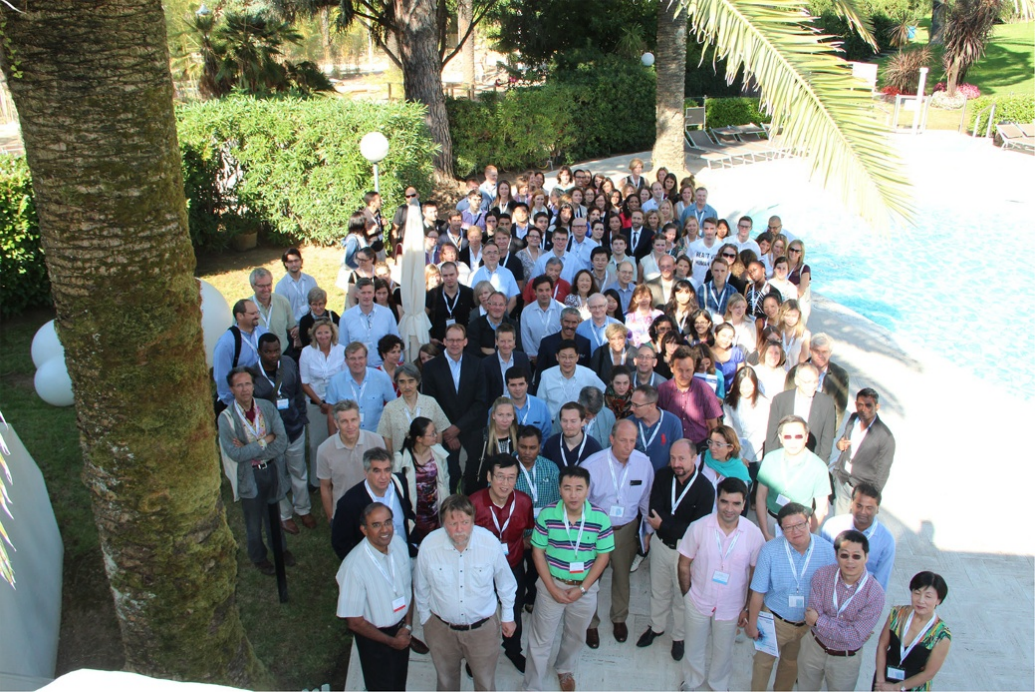
**The Third International Conference on Molecular Neurodegeneration: "Basic biology and disease pathways" - Cannes, France.** The Conference was attended by 179 delegates from around the world to present and discuss the latest research on normal physiological functions and pathological mechanisms of molecular pathways that are relevant to neurodegenerative diseases.

In September 2013, the Third International Conference on Molecular Neurodegeneration: "*Basic biology and disease pathways*" was held in Cannes, France. The three-day conference brought together scientists from around the world to present and discuss the results of their most recent research on the normal physiological functions and pathological mechanisms of molecular pathways that are relevant to neurodegenerative diseases (Figure 1). The current thematic review series in the journal, entitled "***Molecular Neurodegeneration****: Basic Biology and Disease Pathways*", is intended to represent not only the scientific findings presented during the conference, but to also reflect the state-of-the-art in a given field. The review series will consist of a "rolling submission" format in which manuscripts by presenters at the conference will appear at regular intervals in the journal.

The conference covered a wide array of topics concerning neurodegeneration at the genetic, molecular, cellular, physiological, behavioral, and systems levels, and with a translational perspective of moving discoveries from preclinical models into human clinical trials. Sessions discussed the following areas of neurodegeneration research:

-  The genetics of AD and other neurodegenerative diseases
-  The structure and normal physiological functions of molecules involved in neurodegeneration
-  Novel molecular mechanisms of AD, PD, ALS, FTD, DS, and HD
-  Mechanisms of neurotoxicity in PD, AD and other tauopathies
-  Novel therapeutic approaches for neurodegenerative diseases

Molecular mechanisms of neurodegeneration were dominant themes of the conference. Some highlights included novel insights into AD pathogenesis provided by genetic analyses revealing new AD risk factor genes like CD33 and TREM2 involved in innate immunity [1–4]. Deeper knowledge of the structures and normal physiological functions of APP and the α-, β- and γ-secretase enzymes that process APP is assisting the development of disease-modifying AD therapies that target the neurotoxic Aβ peptide [5–9]. Intriguing studies have revealed new molecules involved in neurodegeneration, like C9ORF72 in ALS and FTD, and begin to shed light on their pathogenic roles [10–12]. New insights into the prion-like spread of Aβ and tau pathologies in AD and α-synuclein in PD have also been made [13–15]. Additionally, innovative studies of mechanisms of neurotoxicity have revealed roles for micro-RNAs, transcription factors, and trafficking molecules in AD [16–20]. Novel therapies based on many of these discoveries are also in development, like anti-tau antibodies for AD and other tauopathies [14].

In summary, this review series aims to encapsulate the themes discussed at the Third International Conference on Molecular Neurodegeneration and provide state-of-the-art knowledge of a wide range of fields in neurodegeneration research. It is the hope of the editors that the knowledge conveyed in the review series will provide quality information to readers and stimulate further scientific advances in molecular neurodegeneration that may someday bring an end to these devastating neurodegenerative disorders.

## Abbreviations

Aβ: Beta-amyloid
AD: Alzheimer’s disease
ALS: Amyotrophic lateral sclerosis
APP: Amyloid precursor protein
C9ORF72: Chromosome 9 open reading frame 72
CD33: Cluster of differentiation 33
DS: Down syndrome
FTD: Frontotemporal dementia
HD: Huntington’s disease
PD: Parkinson’s disease
TREM2: Triggering receptor expressed on myeloid cells 2.
